# Quantification of Left Atrial Size and Function in Cardiac MR in Correlation to Non-Gated MR and Cardiovascular Risk Factors in Subjects without Cardiovascular Disease: A Population-Based Cohort Study

**DOI:** 10.3390/tomography8050185

**Published:** 2022-08-31

**Authors:** Charlotte Kulka, Roberto Lorbeer, Esther Askani, Elias Kellner, Marco Reisert, Ricarda von Krüchten, Susanne Rospleszcz, Dunja Hasic, Annette Peters, Fabian Bamberg, Christopher L. Schlett

**Affiliations:** 1 Department of Diagnostic and Interventional Radiology, Medical Center, Faculty of Medicine, University of Freiburg, 79106 Freiburg, Germany; 2 Department of Radiology, Ludwig-Maximilians-University Hospital, 80336 Munich, Germany; 3 Medical Physics, Department of Radiology, Medical Centre, Faculty of Medicine, University of Freiburg, 79106 Freiburg, Germany; 4 Institute of Epidemiology, Helmholtz Zentrum München, German Research Center for Environmental Health, Ingolstädter Landstraße 1, 85764 Neuherberg, Germany; 5 Institute for Medical Information Processing, Biometry and Epidemiology, Medical Faculty, Ludwig-Maximilians-University, 81377 Munich, Germany; 6 German Center for Diabetes Research, München-Neuherberg, 85764 Neuherberg, Germany

**Keywords:** left atrium, cardiovascular risk factors, non-gated, CMR, population-based, cardiovascular magnetic resonance, heart, hypertension, left atrium function, CINE, gated

## Abstract

Background: In magnetic resonance imaging (MRI), the comparability of gated and non-gated measurements of the left atrial (LA) area and function and their association with cardiovascular risk factors have not been firmly established. Methods: 3-Tesla MRIs were performed on 400 subjects enrolled in the KORA (Cooperative Health Research in the Augsburg Region) MRI study. The LA maximum and minimum sizes were segmented in gated CINE four-chamber sequences (LA_max_ and LA_min_) and non-gated T1 VIBE-Dixon (NGLA). The area-based LA function was defined as LAaf = (LA_max_ − LA_min_)/LA_max_. Inter-and intra-reader reliability tests were performed (*n* = 31). Linear regression analyses were conducted to link LA size and function with cardiovascular risk factors. Results: Data from 378 subjects were included in the analysis (mean age: 56.3 years, 57.7 % male). The measurements were highly reproducible (all intraclass correlation coefficients ≥ 0.98). The average LA_max_ was 19.6 ± 4.5 cm^2^, LA_min_ 11.9 ± 3.5 cm^2^, NGLA 16.8 ± 4 cm^2^ and LAaf 40 ± 9%. In regression analysis, hypertension was significantly associated with larger gated LA_max_ (β = 1.30), LA_min_ (β = 1.07), and non-gated NGLA (β = 0.94, all *p* ≤ 0.037). Increasing age was inversely associated with LAaf (β = −1.93, *p* < 0.001). Conclusion: LA enlargement, as measured in gated and non-gated CMR is associated with hypertension, while the area-based LA function decreases with age.

## 1. Introduction

The left atrium (LA) size and function are linked to cardiovascular morbidity and mortality. An enlarged LA and changes in the LA function have a prognostic value in several cardiovascular diseases such as atrial fibrillation [1,2,3,4], heart failure [5,6,7,8], ischemic heart disease [9,10,11] and cardiomyopathy [12,13,14]. Furthermore, an enlarged LA has been shown to be an independent predictor of stroke and death [15,16]. In addition, the LA is progressively becoming a target for structural and electrophysiological interventional procedures, making it an increasingly important subject of interest [17]. Current studies suggest an association of LA measurements with cardiovascular risk factors and prevalent cardiovascular disease [18]. However, associations with risk factors in the subclinical domain have not been firmly established.

The LA size can be measured using different modalities such as echocardiography, computer tomography (CT), or cardiac magnetic resonance imaging (CMR) [17]. The definition of the LA depends on whether the area or the volume is being assessed. CMR is the most accurate technique for non-invasive LA measurement [19]; however, for volumetric analysis, a short axis stack through the LA or gated CINE images in two planes is needed [20]. In everyday clinical practice, CMR is mainly used to measure ventricular volume and function. Hence, the second plane of the LA, which is necessary to determine LA volume, is lacking [21]. It has not yet been established whether LA area measurements derived from readily available four-chamber CINE images are reproducible.

The determination of the LA function in CMR is based on the calculation of LA volumes during various moments in the heart cycle [22]. It remains unclear whether the LA function, as a readily available substitute, can be determined by area-based LA measurements resulting from a single-slice four-chamber CINE view.

Gated CINE images are not available when the clinical focus of the examination is not laid on the heart. Mahabadi et al. quantified the axial LA area in non-contrast-enhanced, ECG-triggered, axial CT images [23] and showed that those measurements were not only readily reproducible, but cardiovascular risk factors correlate with LA size. It is uncertain whether the information obtained from non-gated, axial magnetic resonance imaging (MRI), as is routinely done, can indicate a LA enlargement and prompt further clinical studies.

The objectives of this study are twofold: First, to determine whether the gated and non-gated LA measurements are comparable, and second, whether LA measurements correlate with cardiovascular risk factors in a sample from a population-based study without overt cardiovascular disease.

## 2. Materials and Methods

### 2.1. Study Population and Design

The study sample stems from the cross-sectional KORA-MRI study, which includes *n* = 400 participants that underwent whole-body MRI [24]. Briefly, the KORA-MRI study is a cross-sectional substudy of the KORA-FF4 study (*n* = 2279, enrolled in 2013–2014), which is the second follow-up of the original KORA-S4 study (*n* = 4261, enrolled in 1999–2001). The original KORA-S4 study was designed as a prospective population-based cohort sampled from the city of Augsburg (Southern Germany) and two surrounding counties. For details on the longitudinal design of the KORA studies and the cross-sectional design of the KORA-MRI study, see [24,25]. The main aim of the cross-sectional KORA-MRI study was to use whole-body MRI to ascertain subclinical cardiometabolic disease and identify early related risk factors. Hence, none of the participants of the KORA-MRI study had overt cardiovascular disease. The inclusion criteria for the FF4 follow-up study included agreements to undergo a whole-body MRI examination [24].

**Figure A1:**
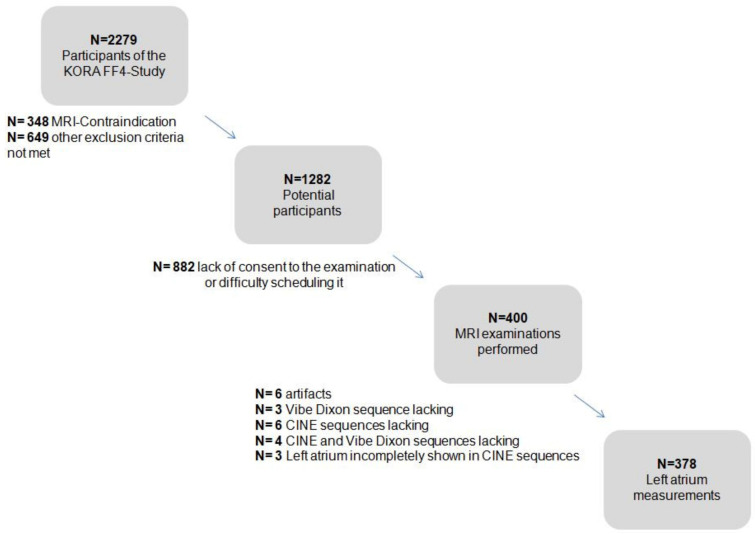
Flow chart depicting the KORA study sample and the cohort design [24,25].

In detail, the MRI exclusion criteria were [24,26]:

- History of cardiovascular disease (myocardial disease, stroke and revascularization therapy);
- Age over 72 years;
- A non-MRI-suitable implant device (cardiac pacemaker or implantable defibrillator, cerebral aneurysm clip, neural stimulator, any type of ear implant, an ocular foreign body, or any implanted device);
- Breast-feeding;
- Claustrophobia;
- A known allergy to gadolinium compounds or renal insufficiency. A detailed flowchart is presented in Appendix A Figure A1.

A mortality and cardiovascular disease morbidity follow-up of the KORA-MRI study is planned; however, the data are not yet available. The study complies with the Declaration of Helsinki, and all participants provided written informed consent. The KORA studies were approved by the ethics committee of the Bavarian chamber of physicians. The institutional review board approved the MRI examination protocol of the medical faculty of Ludwig-Maximilian University Munich. 

### 2.2. Covariates

A large panel of covariates was assessed at the study center examination in a standardized fashion, including interviews, laboratory analysis, health examinations and medication records as detailed elsewhere [24], enabling a comprehensive analysis of a broad range of clinically relevant cardiovascular risk factors. Body surface area (BSA) was calculated using the Du Bois formula (BSA = 0.007184 * body height^0.725^ * body weight^0.425^). The Body Mass Index (BMI) was defined as the body weight in kilograms divided by the squared body height in centimeters. Hypertension was defined as a systolic blood pressure of ≥140 mmHg or a diastolic blood pressure of ≥90 mmHg [27], or the administration of antihypertensive drugs, given that participants were aware of having hypertension. 

The participants‘ diabetes status was assessed based on an oral glucose tolerance test using the 1998 WHO criteria [28] or an established diagnosis of type 2 diabetes. Diabetes was newly diagnosed by a fasting serum glucose ≥ 126 mg/dL or 2–hour serum glucose ≥ 200 mg/dL. Prediabetes was defined as either having a normal fasting glucose concentration and a two-hour serum glucose concentration measured by oral glucose tolerance test in the range of 140–200 mg/dL and/or a fasting glucose level between 110–126 mg/dL.

Participants that met neither of the above-stated definition were labeled as normoglycemic. Smoking status was defined as current smokers, ex-smokers and never-smokers. Laboratory analysis for total cholesterol, high-density lipoprotein (HDL), low-density lipoprotein (LDL) and triglycerides were conducted according to standard protocols. Lipid-lowering medications included statins, fibrates or other lipid-modifying agents. Antihypertensive medication was defined as such only if the compounds taken were classified as antihypertensive by the most recent guidelines [24]. 

### 2.3. Magnetic Resonance Imaging

All participants underwent a whole-body MRI scan during June 2013−September 2014 using a 3-Tesla MRI system (Magnetom Skyra, Siemens AG, Healthcare Sector, Erlangen, Germany) equipped with a whole-body radiofrequency coil-matrix system [24]. For the evaluation of the maximal and minimal LA area using gated images, an unenhanced CINE-steady-state free precession sequence four-chamber view with the following parameters was used: slice thickness 8 mm, voxel size 1.5 × 1.5 mm^2^, the field of view 297 × 360 mm, matrix 240 × 160, repetition time 29.97 ms, echo time 1.46 ms and flip angle 62°, as described previously [24].

For the evaluation of the LA area on a single slice without gating, the two-point T1- weighted opposed phase VIBE-Dixon gradient-echo sequence of the thorax was used with the following parameters: slice thickness 1.7 mm, voxel size: 1.7 × 1.7 mm^2^, the field of view: 488 × 716 mm, matrix 256 × 256 matrix, repetition time: 4.06 ms, echo time: 1.26 × 2.49 ms, with a 9° flip angle [24].

### 2.4. MR-Image Analysis for Left Atrium Size

The LA measurements were conducted by one blinded reader using the medical imaging platform NORA (www.nora-imaging.com, accessed on 15 April 2022).

For the analysis of the LA area, the CINE images and the T1-weighted VIBE-Dixon sequence were manually segmented by an experienced reader blinded to clinical covariates on dedicated offline workstations.

**Figure 1:**
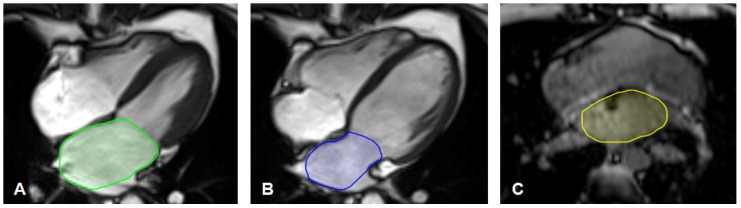
(**A**–**C**) Left atrium segmentation. (**A**) Example of segmentation of the maximal left atrium area (LA_max_) in the gated four-chamber long-axis CINE-sequences in ventricular end-systole just before the opening of the mitral valve.(**B**) Example of segmentation of the minimal left atrium area (LA_min_) in the gated four-chamber long-axis CINE-sequences in ventricular end-diastole just before the closing of the mitral valve.(**C**) Example of segmentation of the non-gated left atrium area (NGLA) using an axial T1 weighted opposed phase VIBE-Dixon gradient echo sequence of the thorax.

For the maximum LA area, the LA was segmented in the four-chamber long-axis view in the ventricular end-systole, just before the opening of the mitral valve (LA_max_; Figure 1A). For the minimal LA area (LA_min_; Figure 1B), the LA was segmented in the ventricular end-diastole just after the closure of the mitral valve [29]. The openings of the pulmonary veins were excluded, and the left atrial appendage was included [30,31].

The non-gated left atrium area (NGLA) was quantified using the T1-weighted VIBE-Dixon sequence of the thorax. In a single slice, the left atrium was manually delineated at the left ventricular outflow tract level and the mitral valve. The pulmonary veins were excluded, and the left atrial appendage was included [23]. An example of segmentation is illustrated in Figure 1C.

As a substitute for the left atrium total ejection fraction (LAtef), which is derived from volume-based LA measurements, we established an area-based measurement, namely the left atrium area fraction (LAaf). The LAaf was calculated with the equation LAaf = (LA_max_ − LA_min_)/LA_max_.

For quality assessment, inter-and intra-reader reliability between analysts was conducted on 31 randomly chosen measurements after at least two months.

### 2.5. Statistical Analysis

For the comparison of LA ascertainment methods, correlations between LA_max_, LA_min_ and NGLA measurements were displayed by scatter plots, and Pearson’s correlation coefficients were provided. Box plots were drawn to show the distribution of the LA area measurements. 

To assess inter-and intra-reader reliability, the intraclass correlation coefficient (ICC) was calculated with an ICC value close to 1, indicating an excellent agreement between the two measurements. Furthermore, the Bland–Altman plots were visually assessed. 

The participants’ descriptive characteristics and cardiovascular risk factors were provided as the mean with standard deviation for continuous variables or percentages and absolute numbers for categorical variables. 

To assess the association between LA measurements and cardiovascular risk factors, univariate linear regression models providing β-coefficients with 95% confidence intervals were conducted to analyze unadjusted associations of demographic data and cardiovascular risk factors with LA-parameters (LA_max_, LA_min_, NGLA and LAaf). 

Furthermore, multivariate regression model analyses were performed to explore the adjusted associations of the combined demographic and cardiovascular risk factors with the LA parameters. The variance inflation factor was used to test for multicollinearity. As a result, only the variable hypertension was used as an umbrella term for the actual systolic and diastolic blood pressure measurement and the intake of antihypertensive medication. LDL was excluded due to multicollinearity. 

A two-sided *p*-value < 0.05 was considered to indicate statistical significance. Statistical analyses were carried out using Stata 16.1 (Stata Corporation, College Station, TX, USA).

## 3. Results

### 3.1. Study Population

Among the 400 participants, 378 were included in the final analysis, while 22 participants were excluded due to the following reasons: in six subjects, imaging artifacts were obscuring an adequate segmentation; in three participants, the VIBE-Dixon sequence was missing; in six participants, the CINE sequences were missing; in four participants both sequences were missing; in three participants, the LA could only be incompletely visualized on the CINE-sequences. A flowchart is presented in Appendix A Figure A1).

In the final sample, the subjects were, on average, 56.3 years old; 218 (57.7%) were male. The average BMI was 28.1 kg/m^2^, while the average body surface measured 1.95 m^2^. Of the subjects, 131 (34.7%) were classified as having hypertension, and 136 (36%) were smokers. A total of 51 (13.5%) subjects had diabetes, and 99 (26.2%) had prediabetes. Additional characteristics of the study sample are summarized in Table 1.

### 3.2. Left Atrium Measurements

Based on the MR measurements, the arithmetic mean of LA_max_ was 19.6 ± 4.5 cm^2^,of LA_min_ 11.9 ± 3.5 cm^2^ and NGLA16.8 ± 4.0 cm^2^ (Table 1). The arithmetic mean for LAaf was 40 ± 9% (Table 1).

**Figure 2:**
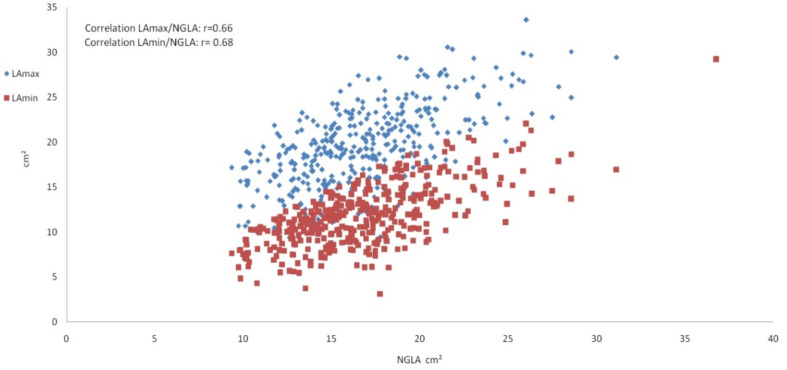
Scatter plot showing the left atrium measurements. Scatter plot depicting the correlation of the maximal left atrium area LA_max_ (blue) and the minimal left atrium area LA_min_ (red) on the *y*-axis with the non-gated left atrium area NGLA on the *x*-axis.

The Pearson correlation coefficient between LA_max_ and LA_min_ was r = 0.87, between LA_max_ and NGLA r = 0.66 (Figure 2) and between LA_min_ and NGLA r = 0.68 (all *p* < 0.001).

### 3.3. Intra- and Inter-Reader Reliability

**Figure A2:**
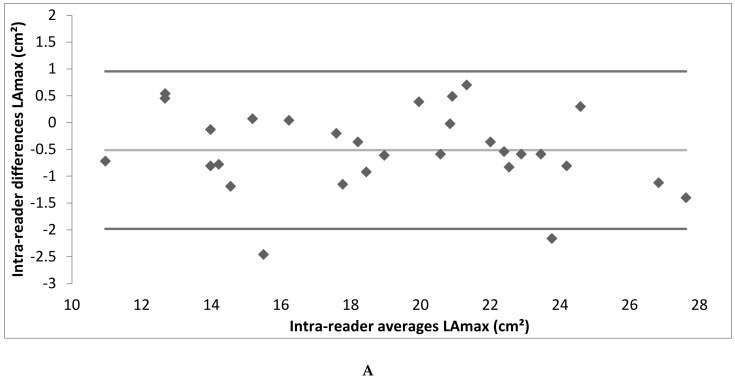

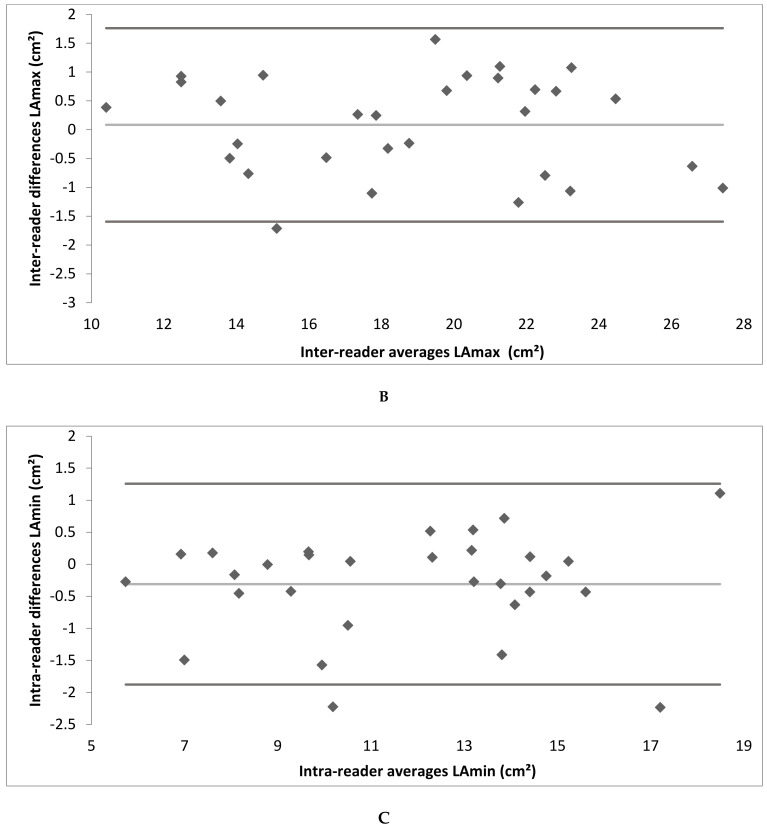

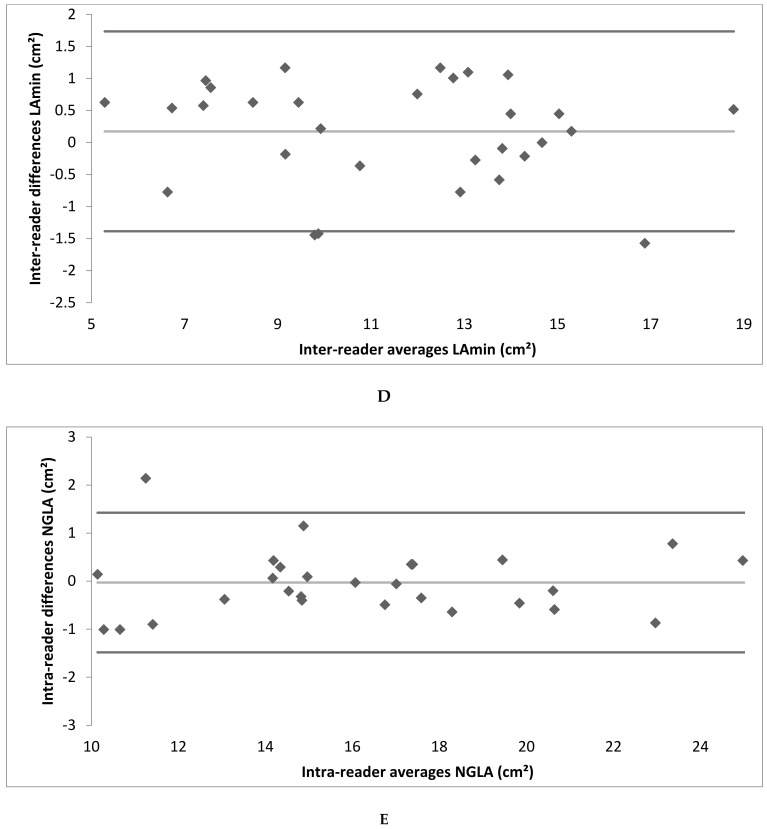

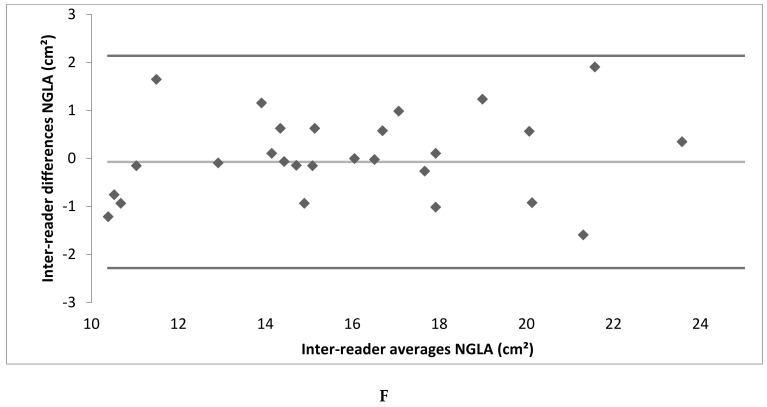
(**A**–**F**) Bland–Altman plot depicting the intra- and inter-reader reliability testing. (LA_max_ denotes maximal gated left atrium area; LA_min_—minimal gated left atrium area; NGLA—non-gated left atrium area from axial slices).

Intra- and inter-reader reliability was excellent. The intra-reader reliability testing resulted in an ICC of 0.99 for LA_max_ and NGLA and an ICC of 0.98 for LA_min_. The inter-reader reliability testing resulted in an ICC of 0.99 for LA_max_, LA_min_ and NGLA (Table 2; the Bland–Altman Plots are detailed in Appendix A Figure A2).

### 3.4. Left Atrium Size in Association with Demographic Data and Cardiovascular Risk Factors

The BSA was significantly positively associated with both gated and non-gated LA in the univariate analysis (Table 3) and remained significant when adjusting for age, sex and cardiovascular risk factors (all *p* < 0.001; Table 4). In the univariate regression analysis BSA was also associated with LAaf (*p* < 0.001; Table 3); however, when adjusting to the other confounders, this association attenuated and was not significant anymore (*p* = 0.13, Table 4).

Age was inversely associated with LAaf in the univariate and multivariate analyses (*p* < 0.001). Furthermore, age was significantly positively associated with NGLA (*p* = 0.008) in the multivariate regression model. In contrast, age was not associated with LA_max_ in any analyses and with LA_min_ only slightly in the univariate analysis (*p* = 0.047, Table 3), which further attenuated in the multivariate analysis (*p* = 0.09, Table 4).

LA_min_, NGLA and LAaf differed significantly with sex in the univariate analysis (all *p* ≤ 0.013, Table 3) but not in the multivariate analysis (Table 4); thus, sex was not independently associated with LA size and function. 

Hypertension was significantly associated with gated and non-gated LA measurements in univariate and multivariate analysis (all *p* ≤ 0.037, Table 3 and Table 4). Effect sizes were larger in LA_max_ than in LA_min_ and NGLA (β = 1.30 (0.22;2.37) vs. β = 1.07(0.26;1.88) vs. β = 0.94 (0.06; 1.82); respectively). Further, hypertension was significantly negatively associated with LAaf in the univariate model (*p* < 0.001); however, when combined with the confounders this association was no longer significant. 

HDL was significantly positively but weakly associated with gated LA_max_ and LA_min_ in multivariate analysis (β = 0.79 and β = 0.50 respectively, *p* ≤ 0.034, Table 4) but showed no significant correlation with NGLA or LAaf. There was no significant association with the remaining blood lipids.

Prediabetes and diabetes were significantly associated with the LAaf in univariate analysis (both *p* ≤ 0.002, Table 3); however, this association did not remain in the multivariate regression model.

Smoking was not associated with LA size or function.

## 4. Discussion

In the present study, we used whole-body MRI to analyze LA measurements from a population-based sample without overt cardiovascular diseases to (1) compare gated and non-gated measurements, (2) assess the association with cardiovascular risk factors. Our findings showed that first, gated and non-gated LA area measurements are readily reproducible with excellent intra- and inter-reader correlation, and second, LA measurements are associated with cardiovascular risk factors, in particular hypertension and age. Consistent associations of BSA and hypertension for both gated and non-gated measurements corroborate the hypothesis that NGLA can readily identify enlarged LA size, which may prompt further clinical investigations and imaging.

Our results thus confirm and extend previous findings. Maceira et al. determined an average of 21 cm^2^ for the maximum LA area measured in the four-chamber view in a small subgroup of 120 normotensive individuals with no known history or risk factors of cardiovascular disease [30], as opposed to the average maximum LA area of 19.6 cm^2^ from our study. This small difference can possibly be explained by the smaller subgroup of subjects in the Maceria et al. study.

CMR provides the possibility of an accurate analysis of the LA function derived from a volume-based LA assessment [22]; however, it is not routinely practiced due to its length of time required and high cost. The area-based function from one single CINE four-chamber view can serve as a substitute for the volume-based left atrium total ejection fraction (LAtef). Previous studies examined subjects without known cardiovascular disease or risk factors and determined an average LAtef of 59 ± 5.8% (*n* = 120), [22] and 60% for males and 61% for females (*n* = 795) [32]. Raisi-Estabragh et al. examined the volume-based LAtef in a large sample of UK Biobank participants with and without cardiovascular risk factors and disease and found an average LAtef of 61.3% [18]. In the population-based MESA (Multi-Ethnic Study of Atherosclerosis) study, subjects with and without atrial fibrillation were examined, resulting in an average baseline LAtef of 44 ± 9 % for 322 subjects without atrial fibrillation and 39 ± 10% for 197 subjects with atrial fibrillation [33]. The area-based average LAaf in our population-based study was 40 ± 9% hence smaller than the LAtef resulting from studies excluding subjects with cardiovascular risk factors and also slightly smaller than the MESA study where subjects with known cardiovascular risk factors were included and the UK Biobank study that included participants with and without cardiovascular risk factor and disease. This could be due to the studied subjects’ demographic differences, or perhaps the area-based LA function underestimates the total ejection fraction. Further studies are required to explore this hypothesis.

Raisi-Estabragh et al. analyzed LA measurements and cardiovascular risk factors, and cardiovascular disease in individuals from the UK Biobank. Their results showed a significant association of CMR-derived LA volume and function with cardiovascular risk factors, which aligns with our findings [18]. 

Multiple studies have shown that maximal and minimal LA size derived from different modalities is significantly associated with BSA [30,34,35,36,37], which was in agreement with our results. This study could show that BSA has further significant links to NGLA. 

Our study showed no significant association between gated and non-gated LA-size and sex in multivariate regression analysis with the confounders. This finding is supported by various other studies that showed that while LA size is generally larger in men, this association does not persist when adjusting for BSA [30,35,37,38,39,40]. 

No independent association between gated LA size and age could be detected, while a significant positive association with NGLA was found. Other published literature [30,41] concluded that normal aging itself does not influence the maximal LA size. In contrast to this, the “MESA” study found a slight enlargement of the LA with aging [40], which was also found by Fredgart et al. using non-contrast-enhanced computer tomography [42] and Singh et al. using echocardiography [43]. Boyd et al. showed that the LA volume increased significantly with age only from the eighth decade [44], while D´Andrea et al. showed that the LA size varies with age starting from the fifth decade [45]. 

Various studies using different imaging modalities have shown that the volume-based LA function was influenced by age [22,46,47,48,49], which is in line with our results that show that age was inversely associated with the area-based LAaf. The Dallas heart study [50] showed that a reduced LAtef was significantly associated with increased mortality in the general population independent of traditional risk factors, while previous studies have shown that reduced LAtef is associated with the risk of developing atrial fibrillation or atrial flutter [51], which underlines the clinical importance of the LA function. We could show that the LAaf is a readily available substitute parameter that might prompt a further clinical and diagnostic workup.

Previous studies showed that the maximal and minimal LA size [18,40,52] is significantly associated with hypertension. Mahabadi et al. could further demonstrate that the LA area from non-enhanced gated axial CT images was significantly associated with hypertension [23]. We could not only show that gated maximal and minimal LA size but also non-gated LA size are positively associated with hypertension. Since an enlarged LA is a well-known risk factor for the development of atrial fibrillation [1,2,4,33], an early diagnosis and control of hypertension may avoid structural LA remodeling and enlargement and ultimately prevent the development of atrial fibrillation. 

While dyslipidemia is known to promote atherosclerosis and hence coronary heart disease [53,54], little is known about the association between blood lipids and LA remodeling. Zemrak et al. [40] detected a significant but weak association of LA volume normalized to BSA with dyslipidemia. At the same time, Raisi-Estabragh et al. showed that the LA volume was smaller in participants with high cholesterol [18]. In our study, HDL was weakly but significantly associated with LA_max_ and LA_min_ in the multivariate regression analysis. However, due to its weak nature, whether this association is of clinical significance remains questionable.

In our study, collective diabetes and prediabetes state were not significantly associated with LA size or function, which is in line with previous studies [23,55].

Our study has certain strengths. First, our sample was from a population-based cohort, and none participants had overt cardiovascular disease. Thus, our results add to knowledge about subclinical cardiovascular disease, which in the long run might be useful to inform preventive strategies. Moreover, high-quality, standardized measurements of a large panel of cardiovascular risk factors enabled comprehensive analyses.

However, our study also has limitations. Limitations include the lack of the LA volume measurements of the same study subgroup for comparison and the study group of overall mainly white ethnicity, which limits generalizability. Furthermore, since all participants were free of overt cardiovascular disease and follow-up for mortality and cardiovascular disease morbidity is not yet available, we were unable to assess the association between LA measurements and hard clinical endpoints. Further efforts are needed to establish the clinical value of LA measurements in this regard. 

## 5. Conclusions

In conclusion, the LA area size measurements are highly reproducible in gated and non-gated CMR, and, as a substitute value, LA area-based function analysis may be performed. In this population-based study, hypertension was independently associated with gated and non-gated LA size. Further, increased age correlated with decreased LA function. Thus, implementing LA size and function evaluation in routine CMR may help prompt further risk assessment.

## Figures and Tables

**Table 1:** Subject characteristics and left atrium measurements. Data are means and standard deviations for continuous variables and counts and percentages for categorical variables.

*n*	378
Age (years)	56.3 ± 9.2
Male sex	218 (57.7%)
BMI (kg/m^2^)	28.1 ± 4.9
Body surface area (m^2^)	1.95 ± 0.22
Systolic blood pressure (mmHg)	120.8 ± 16.8
Diastolic blood pressure (mmHg)	75.3 ± 10.1
Hypertension	131 (34.7%)
Diabetes status	
Normoglycemia	228 (60.3%)
Prediabetes	99 (26.2%)
Diabetes	51 (13.5%)
Smoking status	
Smoker	136 (36%)
Ex-smoker	163 (43.1%)
Never-smoker	79 (20.9%)
Total cholesterol (mg/dL)	218.5 ± 36.8
HDL (mg/dL)	61.8 ± 17.7
LDL (mg/dL)	140.1 ± 33.3
Triglycerides (mg/dL)	133.6 ± 86.2
Lipid-lowering medications	41 (10.9%)
Antihypertensive medications	98 (25.9%)
Left ventricle mass (g) *	141.0 ± 35.3
Left ventricle end-diastolic volume (mL) *	129.6 ± 32.8
Left ventricle end-systolic volume (mL) *	41.0 ± 18.2
Left ventricle stroke volume (mL) *	88.7 ± 20.4
Left ventricle ejection fraction (%) *	69.2 ± 7.8
Maximal left atrium area (cm^2^)	19.6 ± 4.5
Minimal left atrium area (cm^2^)	11.9 ± 3.5
Non-gated left atrium area (cm^2^)	16.8 ± 4
Left atrium area fraction	40 ± 9 %

BMI—body mass index; HDL—high-density lipoprotein; LDL—low-density lipoprotein. * Left Ventricle measurements are based on *n* = 361.

**Table 2:** Results from the inter- and intra-reader reliability testing.

	Inter-Reader Reliability			Intra-Reader Reliability		
	ICC	Relative Difference	Mean Difference	ICC	Relative Difference	Mean Difference
LA_max_	0.99	0.6%	0.09	0.99	2.7%	−0.51
LA_min_	0.99	2.0%	0.18	0.98	3.0%	−0.31
NGLA	0.99	0.3%	−0.07	0.99	0.3%	−0.03

ICC—intraclass correlation coefficient, LA_max_—maximal gated left atrium area; LA_min_—minimal gated left atrium area; NGLA—non-gated left atrium area from axial slices.

**Table 3:** Associations of left atrium measurements with cardiovascular risk factors in separate, univariate models.

	LA_max_		LA_min_		LAaf(%)		NGLA	
	β (95% CI)	*p*	β (95% CI)	*p*	β (95% CI)	*p*	β (95% CI)	*p*
Body surface area (m^2^)	0.85 (0.40; 1.30)	**<0.001**	0.82 (0.47; 1.16)	**<0.001**	−1.72 (−2.63; −0.81)	**<0.001**	1.59 (1.21; 1.96)	**<0.001**
Male sex	0.43 (−0.5; 1.35)	0.37	0.9 (0.19; 1.6)	**0.013**	−3.74 (−5.58; −1.9)	**<0.001**	2.28 (1.5; 3.07)	**<0.001**
Age (years)	−0.18 (−0.64; 0.28)	0.44	0.35 (0.01; 0.7)	**0.047**	−2.20 (−3.10; −1.30)	**<0.001**	0.40 (0.00; 0.80)	0.05
Prediabetes	−0.14 (−1.21; 0.94)	0.80	0.56 (−0.26; 1.38)	0.18	−3.42 (−5.53; −1.3)	**0.002**	0.57 (−0.38; 1.51)	0.24
Diabetes	0.06 (−1.32; 1.44)	0.93	1.01 (−0.04; 2.06)	0.06	−5.14 (−7.87; −2.42)	**<0.001**	1.41 (0.2; 2.62)	**0.023**
Hypertension	1.28 (0.32; 2.23)	**0.009**	1.56 (0.84; 2.28)	**<0.001**	−3.88 (−5.79; −1.97)	**<0.001**	1.78 (0.95; 2.61)	**<0.001**
Ex-smoker	0.56 (−0.48; 1.59)	0.29	0.6 (−0.19; 1.39)	0.14	−1.28 (−3.37; 0.8)	0.23	0.41 (−0.51; 1.32)	0.38
Smoker	−0.12 (−1.38; 1.14)	0.86	−0.25 (−1.21; 0.72)	0.62	1.23 (−1.31; 3.77)	0.34	−0.5 (−1.61; 0.61)	0.38
Total cholesterol (mg/dL)	−0.39 (−0.84; 0.07)	0.10	−0.21 (−0.56; 0.14)	0.23	−0.06 (−0.99; 0.87)	0.90	−0.30 (−0.70; 0.11)	0.15
HDL (mg/dL)	0.20 (−0.26; 0.66)	0.40	−0.05 (−0.4; 0.3)	0.79	1.16 (0.24; 2.08)	**0.014**	−0.49 (−0.89; −0.09)	**0.017**
LDL (mg/dL)	0.37 (−0.49; 1.23)	0.40	−0.09 (−0.75; 0.57)	0.79	2.17 (0.44; 3.9)	**0.014**	−0.92 (−1.67; −0.17)	**0.017**
Triglycerides (mg/dL)	−0.16 (−0.61; 0.3)	0.51	0.10 (−0.25; 0.45)	0.57	−1.08 (−2.00; −0.15)	**0.022**	0.30 (−0.11; 0.70)	0.15
Lipid-lowering medications	0.81 (−0.66; 2.28)	0.28	0.92 (−0.20; 2.05)	0.11	−2.84 (−5.81; 0.13)	0.06	0.93 (−0.36; 2.23)	0.16

LA_max_ denotes maximal gated left atrium area; LA_min_—minimal gated left atrium area; NGLA—non-gated left atrium area from axial slices; LAaf—left atrium area fraction calculated as (LA_max_-LA_min_)/LA_max_; HDL—high-density lipoprotein; LDL—low-density lipoprotein.

**Table 4:** Multivariate regression analysis of the association of left atrium measurements and cardiovascular risk factors and demographic data.

	LA_max_		LA_min_		LAaf(%)		NGLA	
	β (95% CI)	*p*	β (95% CI)	*p*	β (95% CI)	*p*	β (95% CI)	*p*
Body surface area (m^2^)	1.43 (0.76; 2.10)	**<0.001**	1.09 (0.58; 1.59)	**<0.001**	−1.01 (−2.33; 0.31)	0.13	1.78 (1.24; 2.33)	**<0.001**
Male sex	−0.86 (−2.08; 0.36)	0.17	−0.23 (−1.15; 0.69)	0.63	−1.93 (−4.35; 0.48)	0.12	0.29 (−0.71; 1.3)	0.56
Age (years)	−0.13 (−0.66; 0.39)	0.62	0.34 (−0.05; 0.74)	0.09	−1.93 (−2.97; −0.89)	**<0.001**	0.59 (0.16; 1.02)	**0.008**
Prediabetes	−0.86 (−2.02; 0.30)	0.14	−0.34 (−1.21; 0.54)	0.45	−1.18 (−3.48; 1.11)	0.31	−0.88 (−1.83; 0.07)	0.07
Diabetes	−0.71 (−2.32; 0.90)	0.39	−0.10 (−1.32; 1.11)	0.87	−1.84 (−5.03; 1.35)	0.26	−0.22 (−1.54; 1.11)	0.75
Hypertension	1.30 (0.22; 2.37)	**0.018**	1.07 (0.26; 1.88)	**0.010**	−1.20 (−3.32; 0.93)	0.27	0.94 (0.06; 1.82)	**0.037**
Ex-smoker	0.38 (−0.64; 1.39)	0.47	0.34 (−0.43; 1.11)	0.39	−0.45 (−2.47; 1.56)	0.66	−0.08 (−0.92; 0.75)	0.84
Smoker	0.32 (−0.93; 1.57)	0.61	0.17 (−0.78; 1.11)	0.73	0.72 (−1.75; 3.2)	0.57	−0.11 (−1.14; 0.92)	0.83
Total cholesterol (mg/dL)	−0.20 (−0.73; 0.33)	0.46	−0.14 (−0.54; 0.26)	0.51	−0.08 (−1.13; 0.97)	0.88	−0.10 (−0.53; 0.34)	0.66
HDL (mg/dL)	0.79 (0.19; 1.40)	**0.011**	0.50 (0.04; 0.96)	**0.034**	0.31 (−0.89; 1.52)	0.61	0.36 (−0.14; 0.86)	0.16
Triglycerides (mg/dL)	0.05 (−0.58; 0.68)	0.88	0.05 (−0.43; 0.53)	0.83	0.15 (−1.10; 1.40)	0.81	−0.03 (−0.55; 0.49)	0.91
Lipid-lowering medications	0.67 (−0.95; 2.28)	0.42	0.31 (−0.91; 1.53)	0.62	−0.52 (−3.72; 2.67)	0.75	0.30 (−1.03; 1.62)	0.66

LA_max_ denotes maximal gated left atrium area; LA_min_—minimal gated left atrium area; NGLA—non-gated left atrium area from axial slices; LA_af_—left atrium area fraction calculated as (LA_max_-LA_min_)/LA_max_; HDL—high-density lipoprotein.

## Data Availability

The informed consent given by KORA study participants does not cover data posting in public databases. However, data are available upon request by means of a project agreement. Requests should be sent to kora.passt@helmholtz-muenchen.de and are subject to approval by the KORA Board.
